# Improving Monitoring of Indoor RF-EMF Exposure Using IoT-Embedded Sensors and Kriging Techniques

**DOI:** 10.3390/s24237849

**Published:** 2024-12-08

**Authors:** Randa Jabeur, Alaa Alaerjan

**Affiliations:** Department of Computer Science, College of Computer and Information Sciences, Jouf University, Sakaka 72341, Saudi Arabia; rjabeur@ju.edu.sa

**Keywords:** indoor, exposure, RF-EMF, measurements, kriging, downlink, WSN

## Abstract

Distributed wireless sensor networks (WSNs) are widely used to enhance the quality and safety of various applications. These networks consist of numerous sensor nodes, often deployed in challenging terrains where maintenance is difficult. Efficient monitoring approaches are essential to maximize the functionality and lifespan of each sensor node, thereby improving the overall performance of the WSN. In this study, we propose a method to efficiently monitor radiofrequency electromagnetic fields (RF-EMF) exposure using WSNs. Our approach leverages sensor nodes to provide real-time measurements, ensuring accurate and timely data collection. With the increasing prevalence of wireless communication systems, assessing RF-EMF exposure has become crucial due to public health concerns. Since individuals spend over 70% of their time indoors, it is vital to evaluate indoor RF-EMF exposure. However, this task is complicated by the complex indoor environments, furniture arrangements, temporal variability of exposure, numerous obstructions with unknown dielectric properties, and uncontrolled factors such as people’s movements and the random positioning of furniture and doors. To address these challenges, we employ a sensor network to monitor RF-EMF exposure limits using embedded sensors. By integrating Internet of Things-embedded sensors with advanced modeling techniques, such as kriging, we characterize and model indoor RF-EMF downlink (DL) exposure effectively. Measurements taken in several buildings within a few hundred meters of base stations equipped with multiple cellular antennas (2G, 3G, 4G, and 5G) demonstrate that the kriging technique using the spherical model provides superior RF-EMF prediction compared with the exponential model. Using the spherical model, we constructed a high-resolution coverage map for the entire corridor, showcasing the effectiveness of our approach.

## 1. Introduction

Over the past decade, radio technologies have rapidly evolved to meet the increasing demand for connectivity among people and devices, including machines and objects. This surge in wireless communication systems and connected devices has raised concerns about the health impacts of radiofrequency (RF) waves and their perception [1,2]. Given that people spend over 70% of their time indoors, there has been a heightened focus on assessing indoor RF-EMF exposure [3].

Despite the widespread use and low exposure levels, concerns about electromagnetic field (EMF) exposure remain significant [4]. International guidelines, such as those from ICNIRP [5] and IEEE C95.1 [6], have been established to prevent excessive exposure that could impact health.

When electromagnetic (EM) waves propagate from outdoor to indoor environments, power level attenuation can reach up to 20 dB. To mitigate high power attenuation and improve indoor coverage, indoor antennas are often installed, reducing user equipment (UE) power consumption through the uplink power control scheme.

Indoor environments are densely populated with obstructions like furniture, walls, floors, and partitions made of various materials, along with open spaces such as windows and doors. These factors influence the propagation of electromagnetic waves, causing multiple attenuations, reflections, refractions, diffractions, and scatterings, which complicate deterministic assessment of indoor RF-EMF exposure.

Numerous studies have aimed to estimate indoor RF-EMF exposure within the frequency range of 10 MHz to 6 GHz [7,8,9,10,11,12,13]. The most accurate estimation method involves solving Maxwell’s equations using full-wave deterministic techniques, but these methods are impractical for large indoor environments due to high memory load and computational costs [7,8,9]. Ray tracing and ray launching techniques provide good approximations with lower computational demands but are still challenging for unstable environments with unknown dielectric properties and geometries of obstructions. Wireless Sensor Networks (WSNs) are integral to many IoT applications, consisting of numerous sensor nodes that monitor and relay environmental data [14]. These nodes perform various tasks, from critical safety functions to simple data collection, but they all share the core capabilities of data sensing and transmission. By leveraging WSNs, we can significantly expand the monitored areas for RF-EMF exposure, enhancing our ability to supervise and manage several environments effectively. To the best of the authors’ knowledge, no previous work has proposed the use of IoT-embedded sensors based on the Narda NBM-550 and Nucleo-F401RE microcontroller in conjunction with the kriging technique to build a high-resolution, high-accuracy map for monitoring indoor RF-EMF exposure.

The purpose of this work is to design a system leveraging IoT-embedded sensors and Kriging techniques for the efficient monitoring of indoor RF-EMF exposure. To this end, we build a 6LoWPAN network comprising multiple wirelessly connected sensor nodes composed of the Narda NBM-550 and Nucleo-F401RE microcontroller, structured into three virtual layers. The field layer includes the wireless sensors, utilizing various communication channels for extensive coverage and fault tolerance. The data aggregation layer features entities like edge nodes and cloud platforms, which collect and transfer data to higher layers. Finally, the application layer processes the data, making it accessible to end users and handling tasks such as storage, presentation, and formatting. This layered approach ensures efficient data management and utilization. After collecting data using sensor nodes, we apply the kriging technique to characterize and model indoor RF-EMF downlink (DL) exposure, leveraging its strengths in capturing spatial variability, modeling complex indoor environments, and handling irregularly spaced measurement data. Kriging enables accurate interpolation of RF-EMF levels at unsampled locations while also quantifying the associated uncertainty. To achieve this, we first conduct extensive measurements in the corridors of five buildings situated within a few hundred meters of two base stations equipped with multiple cellular antennas (2G, 3G, 4G, and 5G). Subsequently, we identify the optimal semivariogram model to guide the construction of a high-resolution exposure map, providing a detailed spatial representation of RF-EMF levels throughout the entire corridor. The contributions of this work are twofold:

- We design and implement a sensor system based on the Narda NBM-550 and Nucleo-F401RE microcontroller, integrated into a 6LoWPAN wireless network. This system is structured into three virtual layers—Field, Data Aggregation, and Application—to ensure efficient data collection, transmission, and processing for monitoring indoor RF-EMF exposure.
- We apply Kriging techniques to create detailed and accurate spatial maps of RF-EMF downlink exposure in indoor environments. This approach captures spatial variability, models complex indoor environments, and handles irregularly spaced measurement data, providing precise interpolation of exposure levels at unsampled locations.

The remainder of this paper is organized to provide a clear and engaging exploration of the study’s significance and findings. Section 2 delves into related works, highlighting gaps in existing knowledge and positioning this research within the broader scientific context. Section 3 introduces the materials and methods, offering a detailed roadmap of the innovative approach adopted to address these gaps. Section 4 presents a thorough analysis of the results, shedding light on the study’s key contributions and their implications. Finally, Section 5 concludes the paper, emphasizing the importance of this research and outlining its potential to drive advancements in the field.

## 2. Related Works

The assessment of indoor RF-EMF exposure has been a topic of significant research interest, particularly given the increasing reliance on wireless communication technologies using the widely used multiple-input multiple-output (MIMO) [15,16,17,18,19,20] and massive MIMO [21] systems. Theoretical frameworks such as Maxwell’s equations have been fundamental in understanding electromagnetic wave propagation. These equations provide a comprehensive description of how electromagnetic fields interact with different materials and environments, forming the basis for many computational models used in RF-EMF exposure studies. Recent studies have explored various methods for estimating indoor RF-EMF exposure. Full-wave deterministic techniques, which involve solving Maxwell’s equations, are considered the most accurate but are often impractical for large indoor environments due to their high computational demands. Alternative methods, such as ray tracing and ray launching techniques, offer good approximations with lower computational costs. These methods simulate the paths that electromagnetic waves take as they reflect, refract, and scatter within indoor environments, providing valuable insights into exposure levels.

Several studies have explored indoor RF-EMF exposure, highlighting the complexity of accurately assessing exposure levels due to the variability of indoor environments. For instance, research on statistical characterization and modeling of indoor RF-EMF downlink exposure emphasizes the challenges posed by factors like furniture and human movement, using Gaussian distribution models for validation [22]. Reviews on RF-EMF exposure in indoor settings discuss various sources and assessment approaches, noting significant variability across different environments [13]. In ref. [23], the authors review studies on RF-EMF exposure in various confined and occupational environments. They summarize the spatial distribution of RF-EMF exposure in everyday environments across Europe, based on 21 studies conducted between 2000 and 2013. The work in ref. [13] provides an overview of research efforts over the past decade on RF-EMF exposure in indoor environments. It covers different RF-EMF sources, indoor environments, and approaches used to assess exposure. The study highlights that offices and public transport have higher exposure levels compared with homes and apartments. In ref. [24], a study presented the results of measurements and analysis of personal exposure to RF-EMF at both outdoor and indoor school buildings in Spain that is carried out. It focuses on the exposure levels experienced by children and employees who spend significant time in these environments. The findings highlight the variability of RF-EMF exposure in different parts of the school, influenced by factors such as building structure and the presence of wireless communication devices. Additionally, advancements in measurement instruments have been crucial for accurate exposure assessment, aiding epidemiological studies and addressing the challenges of precise measurement. These works collectively underscore the importance of robust modeling and measurement techniques in understanding indoor RF-EMF exposure. Despite these advancements, there are still significant gaps in the literature. One major challenge is the accurate estimation of RF-EMF exposure in large and complex indoor environments, where the dielectric properties of obstructions are often unknown. This uncertainty can lead to significant errors in exposure assessments. Additionally, many existing studies have focused on specific frequency ranges or types of environments, leaving a need for more comprehensive studies that cover a broader range of frequencies and diverse indoor settings.

Methodological approaches in previous studies have varied widely, with each method having its strengths and weaknesses. Full-wave deterministic techniques, while highly accurate, are limited by their computational intensity. Ray tracing and ray launching methods, on the other hand, are more computationally efficient but can struggle with environments where the properties of materials are not well characterized. The kriging technique, which we employ in this study, offers a promising alternative by leveraging spatial statistics to interpolate RF-EMF levels at unsampled locations and quantify the associated uncertainty.

Key findings from recent literature highlight the significant impact of indoor obstructions on RF wave propagation. Materials such as walls, floors, and furniture can cause multiple attenuations, reflections, refractions, diffractions, and scatterings, complicating the deterministic assessment of indoor RF-EMF exposure. Studies have shown that indoor antennas can help mitigate high power attenuation and improve coverage, reducing user equipment (UE) power consumption through uplink power control schemes.

While significant progress has been made in understanding and estimating indoor RF-EMF exposure, there remain substantial challenges and gaps in the literature. By employing the kriging technique, our study contributes to this body of work by providing a high-resolution coverage map that can better predict RF-EMF exposure in indoor environments, thereby addressing the challenges posed by multiple reflections, refractions, diffractions, and scattering.

## 3. Material and Method

### 3.1. Sensor Network Structure

**Figure 1:**
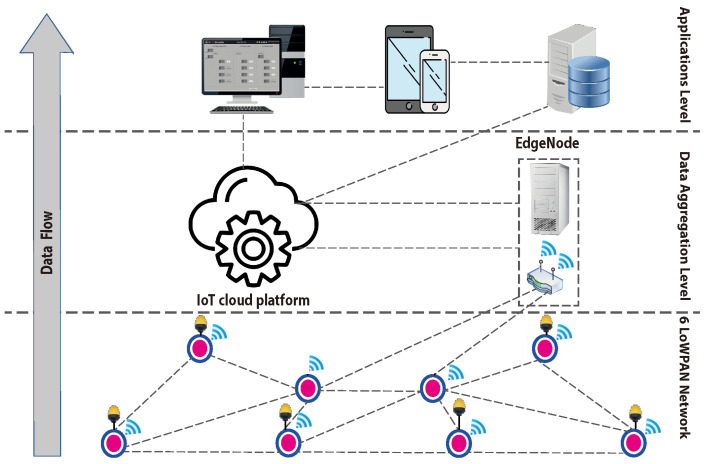
Architecture of considered IoT 6LoWPAN.

Figure 1 illustrates the structure of the wireless sensor network described in this study, specifically a 6LoWPAN network comprising several interconnected sensor nodes. The network architecture is divided into three virtual layers. The first layer, known as the field layer, consists of the wireless sensors within the 6LoWPAN network, which utilize diverse communication channels to achieve broad coverage and enhance fault tolerance. The second layer is the data aggregation layer, which includes components like edge nodes and cloud platforms (such as servers and cloud storage) responsible for gathering data and forwarding it to the upper layers. The third layer is the application layer, where data are processed and made accessible to end users, supporting tasks such as storage, visualization, formatting, and decision-making.

In this configuration, sensor nodes usually connect to the Internet through a single-board router capable of managing multiple data streams. This structure is commonly found in IoT scenarios, such as smart cities. For example, in smart city applications, low-power wireless sensors monitor systems like water distribution and traffic congestion. Due to their limited capabilities, these sensors transmit small data packets, making 6LoWPAN an efficient solution for communication between these devices and their endpoints (e.g., cloud platforms and control centers).

### 3.2. Considered Sensor Node with Narda NBM-550 Broadband Measurement System

In this work, each sensor node dedicated to measurements collection is connected to a Narda NBM-550 broadband field meter [25] with an isotropic EF0691 probe. This probe measures the electric fields for a wide range from 100 kHz to 6 GHz. It covers all the spectrum downlink bands used in Tunisia and is summarized in Table 1.

We emphasize that using the Narda broadband measurement system for spatial measurements to assess downlink exposure poses no risk, given the specific conditions under which the measurements were conducted. Notably, no Wi-Fi access points were present at the measurement locations, and we carefully verified, using the spectrum analyzer (Narda SRM-3006), that no uplink activities occurred during the measurement recordings in the study area. Furthermore, we ensured that no external sources of interference or signal presence, apart from downlink sources, were present. These factors eliminate any potential interference or significant radiation from upstream transmissions that could affect downlink exposure levels. Consequently, the measurements taken are purely representative of downlink emissions. For the sake of real-time measurements collection, we established a reliable communication link using the UART interface to integrate the Narda NBM-550 broadband field meter with the Nucleo-F401RE development board for data transfer. In fact, we connect the NBM-550, known for its precision in measuring electromagnetic fields, to the sensor node, equipped with a Nucleo-F401RE microcontroller and S2LP radio, via the TX and RX pins, ensuring a stable data flow. The STM32CubeMX tool was utilized to configure the UART settings on the Nucleo board, matching the communication parameters of the NBM-550. Firmware is developed using STM32CubeIDE to initialize the UART interface and handle data reception. This setup allows for efficient collection and transmission of electromagnetic field exposure measurements. Using the Narda NBM-550 broadband measurement system, one hundred measurements can be recorded in one minute (i.e., 0.6 s per RF-EMF DL measurement). Through extensive measurements, we show that the spatial averaging periods of three minutes and one minute as used in ref. [22] are very similar. For each measurement location, a sensor node collects three hundred measurements.

### 3.3. Measurements Procedure Description

**Figure 2:**
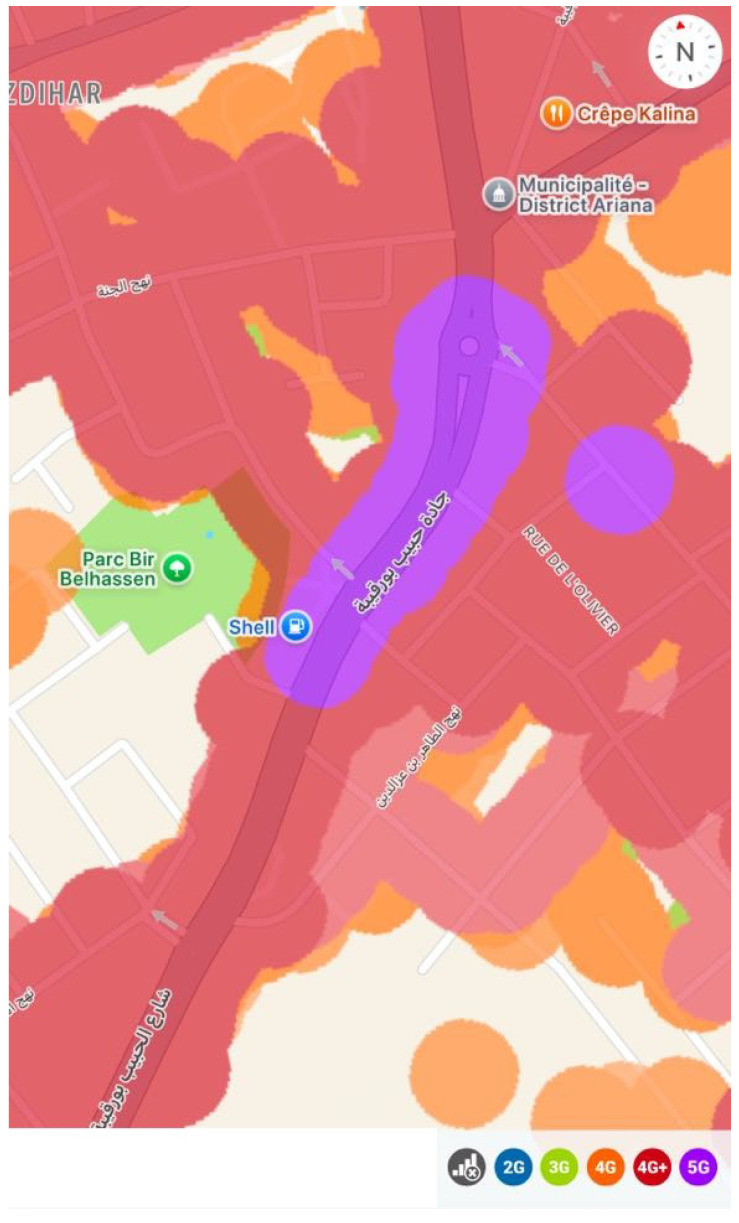
Location of carried-out measurements and available wireless technologies.

Our measurements were conducted using the Narda NBM-550 with Nucleo-F401RE in buildings located in Tunisia, as illustrated in Figure 2. As seen here, the study area is covered by various wireless technologies, including 4G and 5G.

Measurements were conducted at probe heights of 1.7 m as in ref. [22]. We performed a total of 240 spatial measurements across various corridors in multiple buildings, specifically 16 measurement locations per corridor across three corridors in five buildings, maintaining a constant separation distance between each measurement point.

The measurements showed variations in both spatial and temporal domains due to fluctuations in the radio channel and traffic conditions. The quality of the radio channel varies based on the distance to the base station, random environmental changes, and interference, while traffic patterns fluctuate due to user demand and server load. Therefore, an appropriate measurement strategy is essential to account for these variations and to eliminate the influence of mobile data traffic dynamics on the spatial measurements.

To address this, temporal measurements were conducted at a stationary position in an office within one of the buildings to monitor time variations associated with traffic changes over time. These temporal measurements were then used to normalize the spatial measurements, which are influenced by both location and traffic variations.

### 3.4. Field Strength Normalization

In wireless communication systems, traffic impacts the signals emitted by base stations. Given that measurement times at each point differ, so does the associated traffic. This study focuses on analyzing the spatial variations in broadband measurements, necessitating adjustments for traffic variations to ensure consistency. Measurements need to be standardized to a common reference time to accurately attribute observed variations to spatial factors, rather than a combination of spatial and temporal influences. Let $t_{i=1}$ the time for the initial measurement point. It will be used as reference time. To standardize each field measurement at point *i* to the reference time $t_{i=1}$, we adjusted it by a correction factor, $\frac{E_{ref}\left(t_{i=1}\right)}{E_{ref}\left(t_{i}\right)}$. Thus, the equivalent value can be written as [22]

(1) $$E_{t_{i}\_eq}\left(t_{i=1}\right)=E\left(t_{i}\right)\times \frac{E_{ref}\left(t_{i=1}\right)}{E_{ref}\left(t_{i}\right)}.$$

For each measurement point, we compute the mean of measurements performed in three minutes.

### 3.5. Kriging for RF-EMF Exposure Interpolation

**Figure 3:**
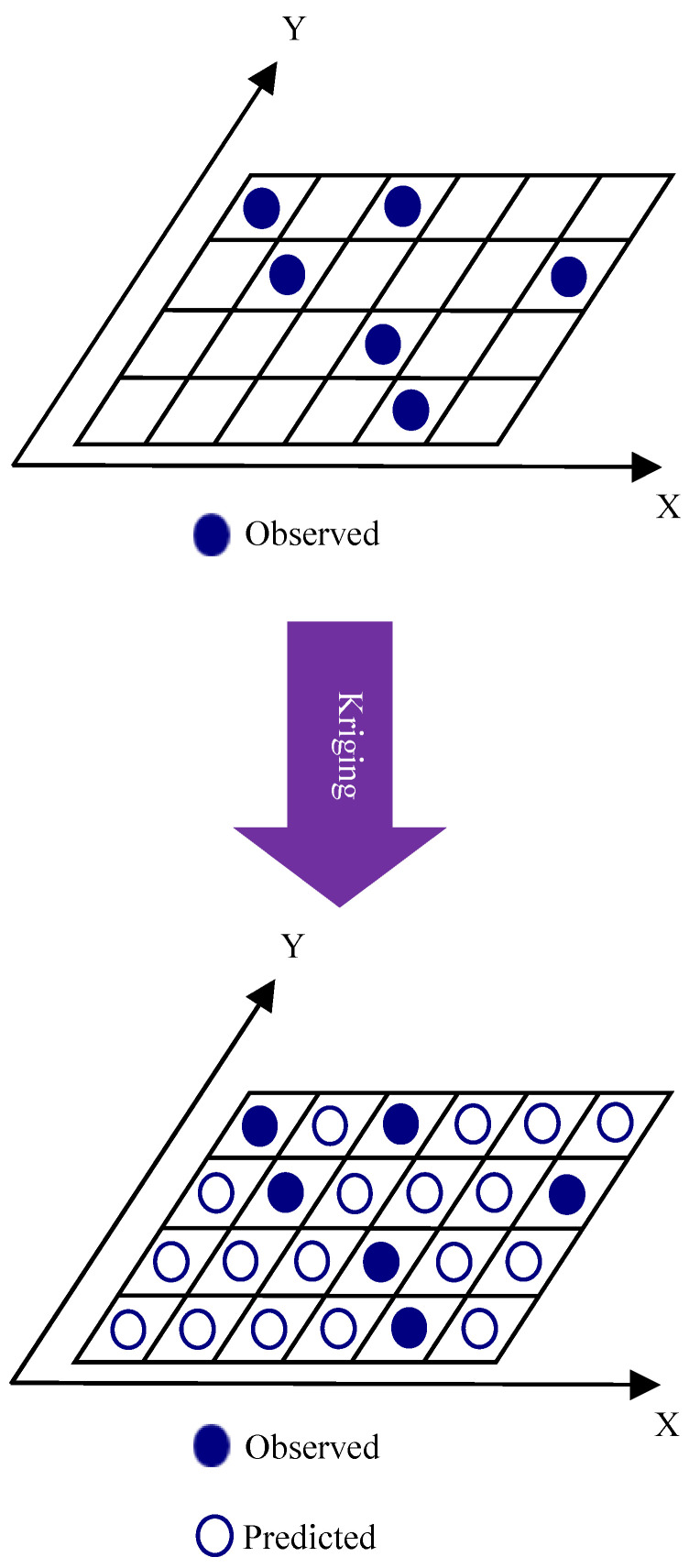
Kriging interpolation technique.

Kriging is a geostatistical technique used for spatial interpolation, particularly in the field of spatial statistics and geostatistics. It is often employed to estimate values at unmeasured locations based on observed values at sampled locations [26,27,28,29]. Kriging is a highly suitable method for predicting indoor RF-EMF exposure due to its ability to account for spatial variability, model complex indoor environments, and handle irregularly spaced data points. By leveraging the spatial correlation between measurement locations, kriging can accurately interpolate RF-EMF levels at unsampled points as shown in Figure 3, providing not only predictions but also quantifying the associated uncertainty. This is crucial in environments where multiple sources and frequencies contribute to exposure, as kriging can be adapted to incorporate these factors, resulting in more comprehensive and reliable assessments. Additionally, kriging’s successful application in other environmental exposure studies underscores its effectiveness in managing spatially variable data, making it an excellent choice for indoor RF-EMF mapping.

A critical component of kriging is the variogram, γ(h), which characterizes the spatial dependence between data points. The variogram is a function of the lag distance *h*, which is the distance between pairs of data points. The variogram models the degree of similarity or dissimilarity between data values separated by a given distance *h*. The variogram is often expressed as [30]

(2) $$\gamma \left(h\right)=\frac{1}{2}E\left[{\left(E\left(l\right)-E\left(l+h\right)\right)}^{2}\right],$$

where E(l) and E(l+h) are values at points separated by distance *h*.

Two common variogram models used within kriging are the exponential model and the spherical model. The exponential model is a popular choice in kriging when spatial correlation decreases exponentially with distance. The variogram, which measures the spatial dependence between points, is used to model the spatial correlation structure. In the exponential model, the variogram is described by an exponential function. The mathematical representation of the variogram for the exponential model is often given as [30]

(3) $$\gamma \left(h\right)=c\times \left(1-\exp\left(-\frac{h}{a}\right)\right),$$

where *c* is the sill (maximum variability), *a* is the range (distance at which correlation becomes negligible), and *h* is the separation distance between locations. The spherical model is another commonly used model in kriging, especially when there is a clear cutoff point beyond which spatial correlation becomes negligible. The variogram for the spherical model is represented by a spherical function given by [30]

(4) $$\gamma \left(h\right)=\left\{\begin{array}{cc} c\times \left(1.5\times \left(\frac{h}{a}\right)-0.5\times {\left(\frac{h}{a}\right)}^{3}\right) & \mathrm{if}\;h\leq a\\ c & \mathrm{if}\;h>a \end{array}\right.$$

For h≤a, the variogram increases with the lag distance *h*. The specific form of the spherical variogram within this range represents a smooth increase from the origin (where h=0) to the range *a*, beyond which the variogram levels off and remains constant at the sill value *c*.

For h>a, the variogram becomes constant and equals *c*. This indicates that beyond the range *a*, the spatial correlation between data points does not increase with distance; the points are essentially uncorrelated, and the variance remains at the sill value.

The choice between these models depends on the characteristics of the spatial data, and model selection is often guided by empirical analysis of the variogram.

**Figure 4:**
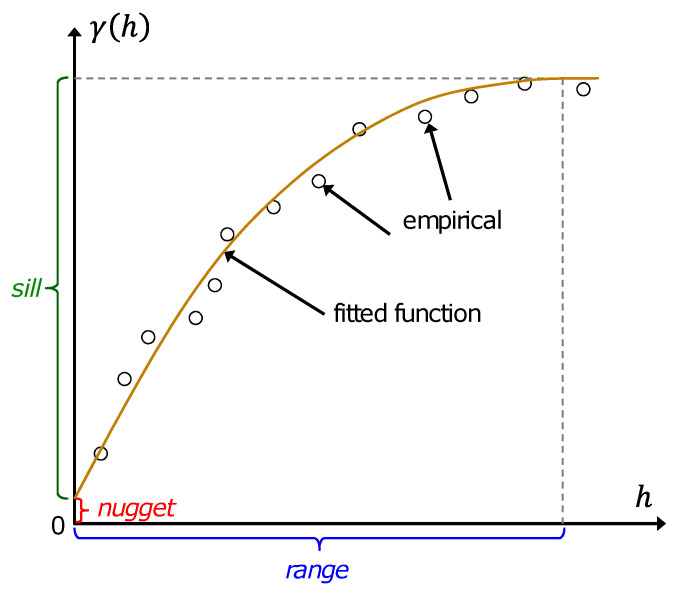
Variogram parameters.

In Figure 4, we illustrate the three key parameters of a variogram. The first is the nugget, which indicates the discontinuity at the origin due to measurement errors. The second parameter, known as the sill, corresponds to the maximum semivariance, equivalent to the process variance. Lastly, the range defines the distance at which two samples lose correlation, causing the variogram to reach the sill. Additionally, it can be observed that at the same location, the semivariance value is zero, meaning γ(0)=0.

Kriging operates as a best linear unbiased estimator, where the predicted value $\hat{E}\left(l_{0}\right)$ at a location $l_{0}$ is expressed as a weighted sum of the observed values $E\left(l_{1}\right),E\left(l_{2}\right),\ldots ,E\left(l_{n}\right)$.

(5) $$\hat{E}\left(l_{0}\right)=\sum_{i=1}^{n}\lambda_{i}E\left(l_{i}\right).$$

The kriging weights $\lambda_{i}$ are determined by minimizing the estimation variance subject to the constraint of unbiasedness. This leads to the following system of linear equations in matrix form:

(6) $$\left(\begin{array}{cc} \Gamma & 1\\ 1^{T} & 0 \end{array}\right)\left(\begin{array}{c} \lambda \\ \mu \end{array}\right)=\left(\begin{array}{c} \gamma_{0}\\ 1 \end{array}\right),$$

where Γ is an n×n matrix of variogram values between data points, 1 is an n×1 vector of ones, and $\gamma_{0}$ is an n×1 vector of variogram values between each data point and the prediction location $l_{0}$, λ is an n×1 vector of kriging weights, and μ is a Lagrange multiplier ensuring the unbiasedness constraint $\sum_{i=1}^{n}\lambda_{i}=1$.

Solving this system yields the kriging weights, which are used to compute the predicted value at $l_{0}$. The kriging variance, which quantifies the uncertainty of the prediction, is given by:

(7) $$\sigma_{K}^{2}\left(l_{0}\right)=\gamma \left(l_{0},l_{0}\right)-\lambda^{T}\gamma_{0}+\mu .$$

This approach allows for accurate spatial predictions while providing an associated measure of uncertainty.

### 3.6. Metric of Evaluating the Constructed Variogram Model

Consider *N* the number of performed measurements in a given corridor; the Root Mean Square Error (RMSE) for a given variogram model can be expressed as

(8) $$\mathrm{RMSE}={\left(\frac{1}{\mathrm{card}\left(N\right)}\sum_{l\in N}{\left(\hat{E}\left(l\right)-E\left(l\right)\right)}^{2}\right)}^{\frac{1}{2}}.$$

where $\hat{E}\left(l\right)$ and $E\left(l\right)$ are the estimated and real E fields, respectively.

## 4. Results and Discussions

**Figure 5:**
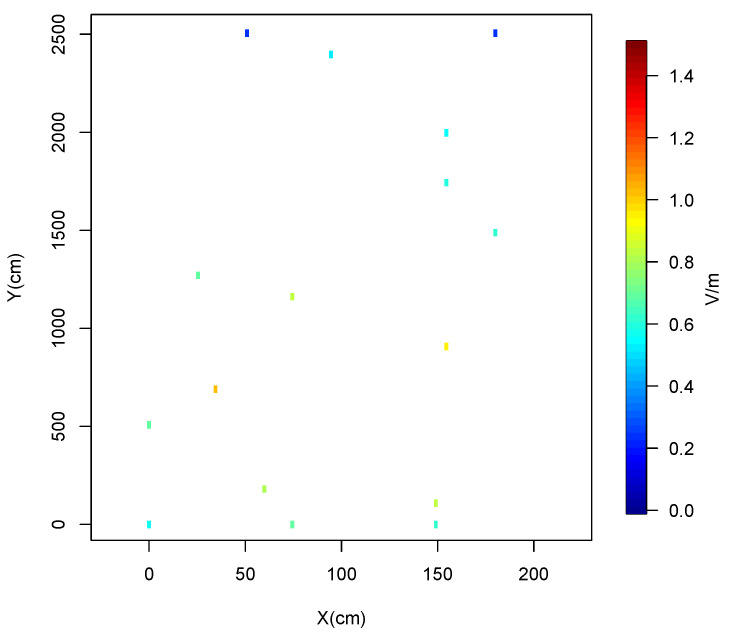
Real RF-EMF measurements.

To characterize the indoor RF-EMF DL exposure in the corridors of considered buildings, we utilize the Kriging technique, as described in Section 3.5. Initially, we conduct measurements in several corridors using the proposed measurement system based on the Narda NBM-550 in conjunction with the Nucleo-F401RE. Figure 5 illustrates the actual RF-EMF measurements taken in Corridor 1.

**Figure 6:**
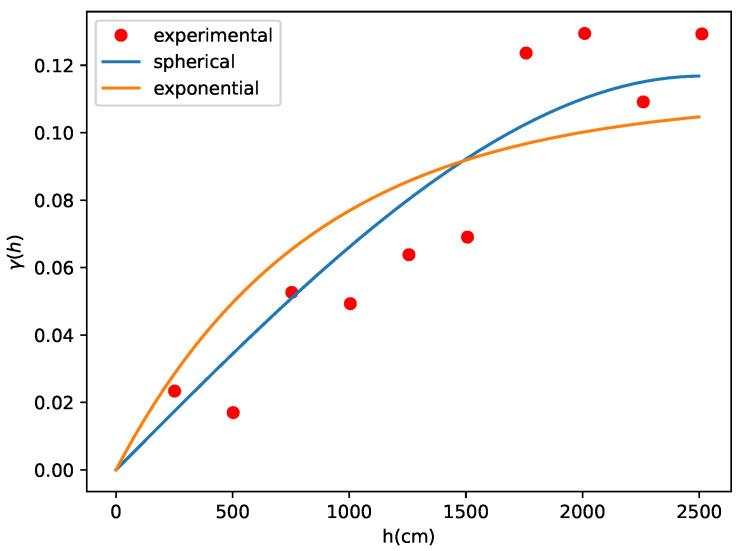
Semivariogram.

From these measurements, we generated the corresponding semivariogram using the Kriging technique, comparing both the spherical and exponential models. Figure 6 indicates that the spherical model provides a superior fit compared with the exponential model. To substantiate this observation, Table 2 presents a performance comparison between Kriging using the spherical model and the exponential model. The data clearly demonstrate that the spherical model outperforms the exponential model, leading to a more accurately constructed map.

**Figure 7:**
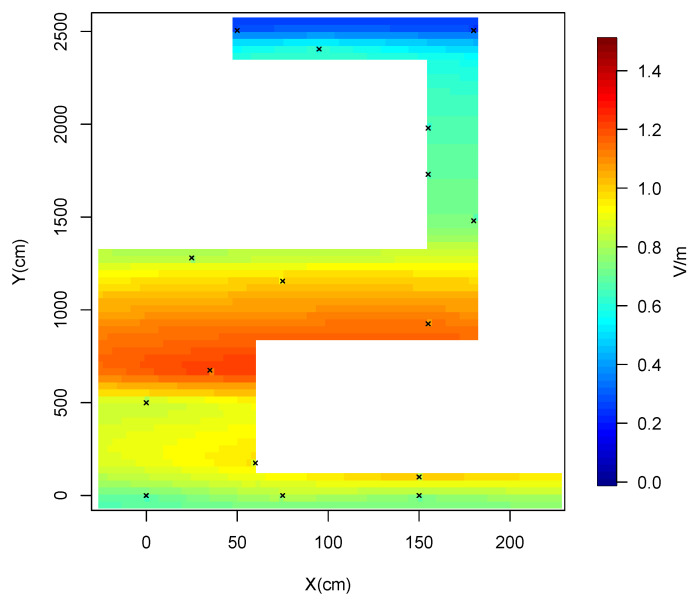
Constructed map using the spherical model.

Consequently, we adopted the spherical model for constructing the high-resolution map of the entire corridor. This map is displayed in Figure 7. It is shown that the maximum average RF-EMF DL exposure levels at various positions within the corridor are well below the limits established by the International Commission on Non-Ionizing Radiation Protection (ICNIRP) [31].

## 5. Conclusions

This study proposes the use of WSN to monitor the indoor RF-EMF exposure induced by cellular networks. To this end, we first proposed a measurement system based on the Narda NBM-550 and Nucleo-F401RE microcontroller board. The aim is to characterize and model indoor RF-EMF DL exposure using the collected measurements and kriging technique. First, several indoor measurements are conducted in an area covered by various frequency bands, including those used for 5G. By comparing the spherical and exponential models, we demonstrated that the spherical model provides a superior fit for predicting RF-EMF exposure levels. The high-resolution coverage map constructed using the spherical model revealed that the maximum average RF-EMF DL exposure levels within the corridor are well below the limits established by the ICNIRP. These findings underscore the effectiveness of the kriging technique in accurately modeling and predicting RF-EMF exposure in complex indoor environments.

For future work, several avenues can be explored to enhance the understanding and assessment of indoor RF-EMF exposure. Firstly, expanding the measurement campaign to include a wider variety of indoor environments, such as residential buildings, offices, and public transport, would provide a more comprehensive dataset. Additionally, incorporating temporal variations by conducting long-term measurements could offer insights into the fluctuations of RF-EMF exposure over time. Furthermore, integrating advanced machine learning algorithms with the kriging technique could improve the accuracy and efficiency of exposure predictions. Another important research axis involves the analysis of measurement uncertainty, which is planned for future investigation. For large-scale deployments, the Narda NBM-550 can be replaced with frequency-selective equipment such as the ExpoM-RF4, MVG EME Spy Evolution, or Narda SRM-3006. This substitution ensures that only downlink bands are considered and enables the reconstruction of RF-EMF exposure maps for each frequency band, facilitating a more detailed assessment by frequency band. Finally, investigating the impact of emerging wireless technologies, such as beyond 5G, on indoor RF-EMF exposure will be crucial as these technologies become more widespread. These future directions will contribute to a more thorough understanding of indoor RF-EMF exposure and help address public concerns regarding wireless communication systems.

## Figures and Tables

**Table 1:** Spectrum downlink bands in Tunisia.

Bands	Ranges (MHz)
GSM 900	925–960
GSM 1800	1805–1880
UMTS 2100	2110–2170
4G LTE 800	791–821
4G LTE 1800	1805–1880
4G LTE 2600	2620–2690
5G 3500	3300–3800

**Table 2:** Comparision between RMSE produced by the kriging technique using both spherical and exponential models.

	Spherical	Exponential
RMSE (V/m)	0.016	0.023

## Data Availability

The data presented in this study are available on request from the corresponding author.
